# Cariprazine, A Dopamine D_2_/D_3_ Receptor Partial Agonist, Modulates ABCG2-Mediated Multidrug Resistance in Cancer

**DOI:** 10.3390/cancers10090308

**Published:** 2018-09-04

**Authors:** Noor Hussein, Charles R. Ashby, Haneen Amawi, Angelique Nyinawabera, Atul Vij, Vishwa M. Khare, Chandrabose Karthikeyan, Amit K. Tiwari

**Affiliations:** 1 Department of Pharmacology and Experimental Therapeutics, College of Pharmacy & Pharmaceutical Sciences, University of Toledo, Toledo, OH 43614, USA; noor.hussein@rockets.utoledo.edu (N.H.); haneen.amawi@rockets.utoledo.edu (H.A.); angelique.nyinawabera@rockets.utoledo.edu (A.N.); atulvij1994@yahoo.com (A.J.); 2 Department of Pharmaceutical Sciences, College of Pharmacy, St. John’s University, Queens, NY 11439, USA; cnsratdoc@optonline.net; 3 Cell and Developmental Biology, School of Medicine, University of Pennsylvania, Philadelphia, PA 19104, USA; khare_vm@yahoo.com; 4 Department of Pharmacy, Indira Gandhi National Tribal University, Lalpur, Amarkantak, MP 484887, India; karthinobel@gmail.com

**Keywords:** ABCG2, Cariprazine, Colon cancer, Lung cancer, Dopamine D_3_-preferring D_2_/D_3_ receptor partial, Multidrug resistance

## Abstract

Multidrug resistance (MDR) is a continuing clinical problem that limits the efficacy of chemotherapy in cancer. The over expression of the ATP-binding cassette (ABC) family G2 (ABCG2) transporter is one of the main mechanisms that mediates MDR in cancer. Molecular modeling data indicated that cariprazine, a dopamine D_2_/D_3_ receptor partial agonist, had a significant binding affinity for ABCG2 transporter with a Glide XP score of −6.515. Therefore, in this in vitro study, we determined the effect of cariprazine on MDR resulting from the overexpression of ABCG2 transporters. Alone, cariprazine, at concentrations up to 20 μM, did not significantly decrease cell viability. Cariprazine, at concentrations ranging from 1 to 10 μM, did not significantly alter the cytotoxicity of mitoxantrone (MX) in the parental non-small cell cancer cell line, H460 and colon cancer cell S1. However, cariprazine (1–20 μM) significantly enhanced the efficacy of ABCG2 substrate antineoplastic drug MX in the ABCG2-overexpressing MDR cell line, H460-MX20 and S1M1-80, by reducing the resistance fold from 28 to 1 and from 93 to 1.33, respectively. Cariprazine, in a concentration-dependent (1–20 μM), significantly increased the intracellular accumulation of Rhodamine 123 in S1M1-80. Interestingly, 10 or 20 μM of cariprazine significantly decreased the expression levels of the ABCG2 protein in the colon and lung cancer cell lines, suggesting that cariprazine inhibits both the function and expression of ABCG2 transporters at nontoxic concentrations. Overall, our results suggest that cariprazine, via several distinct mechanisms, can resensitize resistant cancer cells to mitoxantrone.

## 1. Introduction

Multidrug resistance (MDR) is one of the primary mechanisms that significantly decreases or abolishes the efficacy of cancer chemotherapy [1]. MDR is defined as the resistance of cancer cells to structurally and mechanistically unrelated compounds [2,3]. It is well known that the overexpression of specific ATP-binding cassette (ABC) proteins in the cellular membrane of cancer cells can mediate MDR [4]. The ABC transporter superfamily consists of seven subfamilies, ABC-A to G, based on amino-acid sequence [5]. Currently, there are 48 known human ABC transporters that have been identified [6]. Three human ATP binding cassette (ABC) transporters, including, ABCB1 (P-glycoprotein), ABCC1 (multidrug resistance protein 1) and ABCG2 (breast cancer resistance protein) have been implicated in mediating MDR through facilitating the efflux of a wide range of structurally unrelated therapeutic molecules, which either greatly reduces or abolishes their efficacy [7].

ABCG2 is a 72 kDa half-transporter that has only one nucleotide binding domain (NBD) and one transmembrane binding domain (TMD) [8]. In order for it to be functionally active, the ABCG2 must be dimerized [9,10]. The in vitro and in vivo overexpression of the ABCG2 transporter has been reported to mediate MDR in cancer cells by catalyzing the efflux of various anticancer drugs, including mitoxantrone (MX), anthrapyrazole, topotecan, irinotecan and SN-38 [11]. Thus, it was hypothesized that compounds, which inhibit the efflux function of the ABCG2 transporter, should decrease the efflux of anticancer compound substrates, thereby increasing their intracellular concentration and subsequently, their efficacy [12]. Indeed, numerous cellular and animal studies have shown that numerous compounds, e.g., fumitremorgin C (FTC) [13], K0143 [14] tariquidar [15], nilotinib [16] and CCTA-1523 [17] can increase the efficacy of certain anticancer drugs in cancer cells where MDR is mediated by the overexpression of ABCG2 transporters. However, currently, there is no clinically approved ABCG2 modulator for treating MDR in cancers.

Pharmacophore modeling studies indicate that for optimum binding of substrates to the ABCG2 transporter, the following features should be present: one hydrogen bond acceptor and two hydrophobic centers [18]. Therefore, based on these features, we previously postulated that the D_3_ dopamine receptor antagonists PG01037, NGB2904, SB-277011A, and U99194, should have significant interactions with the ABCG2 transporter [19]. There were data suggesting that certain dopamine D_3_ receptor antagonists have specific structural features such as a carboxamide moiety, and at least two hydrophobic centers and a long molecular axis that allows binding with moderate to high affinity to ABCG2 transporters [18]. Indeed, we reported that the above listed dopamine D_3_ receptor antagonists significantly reversed ABCG2-mediated MDR to antineoplastic drugs in lung (i.e., H460 and H460-MX20) and colon cancer cell lines (i.e., S1 and S1-M180) overexpressing ABCG2 transporters [19]. Furthermore, the rank order of potency for PG01037, NGB2904, SB-277011A, and U99194 to reverse resistance to mitoxantrone in the cancer cells overexpressing ABCG2 transporters was similar to the rank order of the docking scores for the ABCG2 binding, which were −9.704, −9.299, −9.068, and −6.525, respectively [19]. Therefore, based on the above results, we postulated that cariprazine, a dopamine D_3_-preferring D_2_/D_3_ receptor partial agonist, may interact with the ABCG2 transporter and modulate ABCG2-mediated MDR, as similar to PG01037, NGB2904, and SB277011A, it has a carboxamide moiety, at least two hydrophobic centers and a long molecular axis [20]. Therefore, in this study, we determined the in vitro efficacy of cariprazine to reverse resistance to the anticancer drug, mitoxantrone, an ABCG2 substrate, mediated by the overexpression of ABCG2 transporters in non-small lung cancer cells and colon cancer cells. We also conducted experiments to gain insight into the mechanisms by which cariprazine reverses resistance to mitoxantrone.

## 2. Results

### 2.1. Cariprazine Significantly Increases the Efficacy of Mitoxantrone in Cancer Cell Lines Overexpressing the ABCG2 Transporter

**Figure 1:**
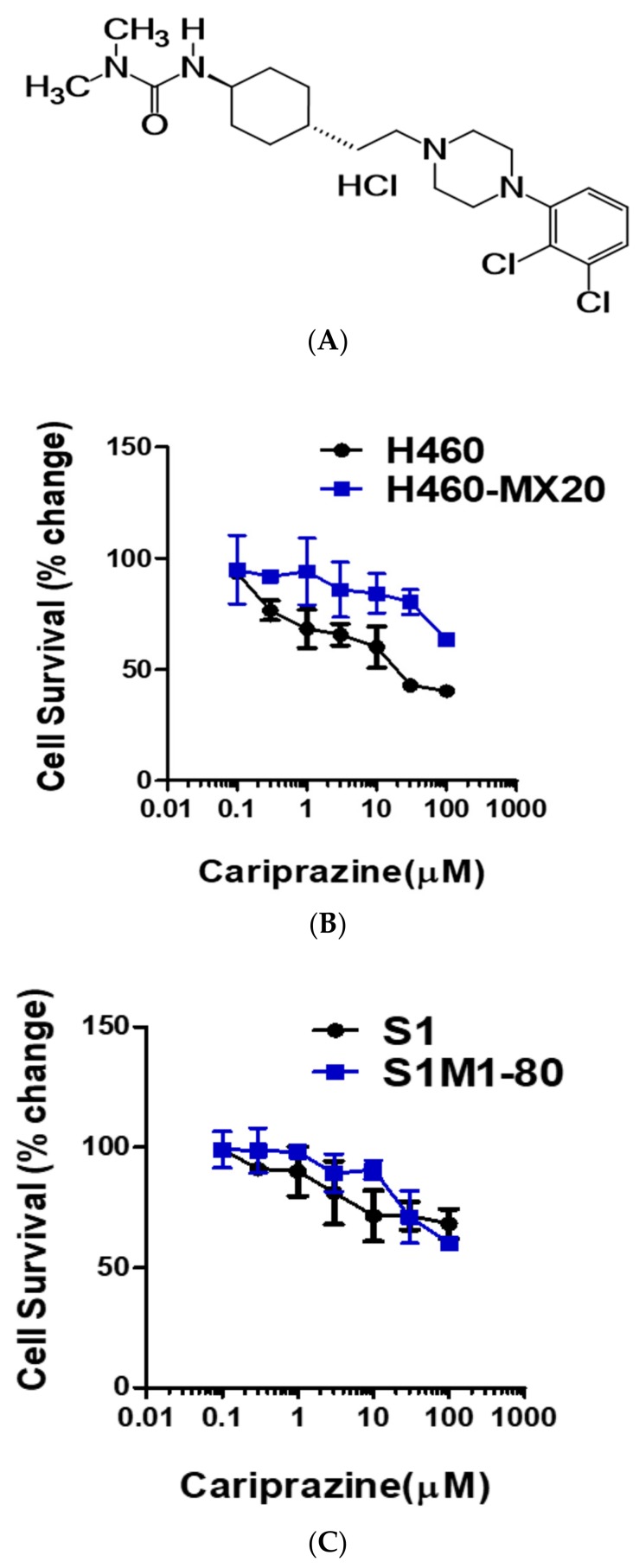
(**A**) Chemical structure of cariprazine. (**B**) 3-(4,5-Dimethylthiazol-2-yl)-2,5-diphenyltetrazolium bromide (MTT) cytotoxicity assay results are shown for H460 and H460-MX20 cells incubated with cariprazine and (**C**) S1 and S1M1-80 cells incubated with cariprazine. The data points represent the mean ± SD of at least three independent experiments repeated in triplicate.

Selecting the appropriate pairs of MDR-ABC transporter overexpressing (i.e., resistant cell lines) and non-resistant cell lines and determining if the drugs being tested significantly affect cell viability are crucial steps in determining if cariprazine can surmount ABCG2-mediated resistance. Thus, we chose the drug-selected cell line pairs, H460-MX20, and S1M1-80, with S1 and H460 being the parental, nonresistance cell lines. We then determined the effect of the cariprazine alone on cell viability. As shown in Figure 1B,C, cariprazine did not produce significant cytotoxicity at 20 μM in either H460 or H460/MX20 cells, or S1 and S1M1-80 cells. Based on these results, we used 20 μM of cariprazine as the highest concentration in all subsequent experiments.

**Figure 2:**
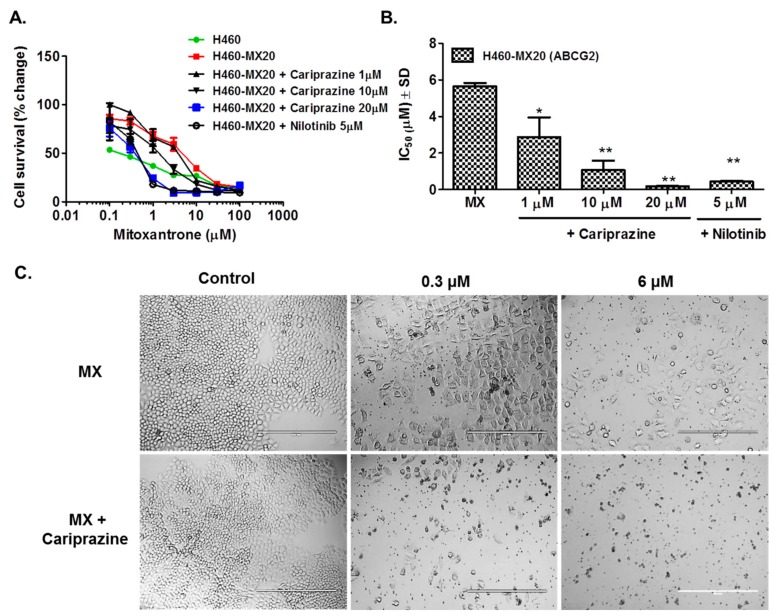
The effect of cariprazine on the efficacy of MX in the ABCG2 overexpressing cell line H460-MX20. (**A**) MTT cytotoxicity is expressed as the percentage change in cell survival. (**B**) The IC_50_ values for MX alone (control) or in combination with different concentrations of cariprazine or with 5 μM of nilotinib. (**C**) Microscopic images illustrating the synergistic effects of cariprazine on the efficacy of MX at different concentrations. Scale bar: 20×. The data points represent the means ± SD of at least three independent experiments, each with triplicate determinations. * *p* < 0.05; ** *p* < 0.01 vs. control cells incubated with MX alone.

**Figure 3:**
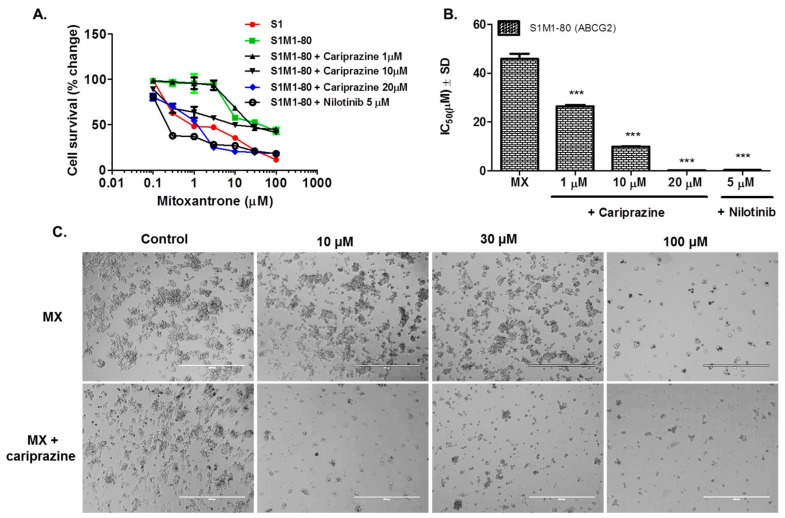
The effect of cariprazine on the efficacy of MX in the ABCG2 overexpressing cell line S1M1-80. (**A**) MTT cytotoxicity is expressed as the percentage change in cell survival. (**B**). The IC_50_ values for MX alone (control) or in combination with different concentrations of cariprazine or with 5 μM of nilotinib in S1M1-80 cells. (**C**) Microscopic images illustrating the synergistic effects of cariprazine on the efficacy of MX at different concentrations. Scale bar: 20×. The data points represent the means ± SD of at least three independent experiments, each with triplicate determinations. *** *p* < 0.001 vs. control cells incubated with MX alone.

We determined the efficacy of 1, 10, or 20 μM of cariprazine on the H460 and H460/MX20 cells and S1, S1M1-80 cells, to surmount ABCG2-mediated MDR to MX. As shown in Table 1 and Figure 2A,B, cariprazine, at 1, 10, or 20 μM, significantly increases the efficacy of MX in ABCG2-overexpressing H460/MX20cells; similarly, 1, 10, or 20 μM of cariprazine significantly increases the efficacy of MX in the ABCG2-overexpressing S1M1-80 cells. Thus, cariprazine reverses the MDR mediated by overexpression of the ABCG2 transporter (Table 2, and Figure 3A,B). As previously reported, the IC_50_ values of MX in the H460-MX20 and S1M1-80 cell lines were significantly reduced by combining 5 μM of nilotinib (an inhibitor of ABCB1 and ABCG2) [21] with MX. However, cariprazine did not significantly alter the cytotoxicity of MX in the parental cell lines, H460 and S1.

After the incubation of 10 or 20 μM cariprazine and MX (0.3–6 μM), changes in cell morphology, including significant reduction in average cell number, confluence, volume and area were observed (Figure 2C and Figure 3C).

### 2.2. Cariprazine Synergistically Increases the Efficacy of MX

**Figure 4:**
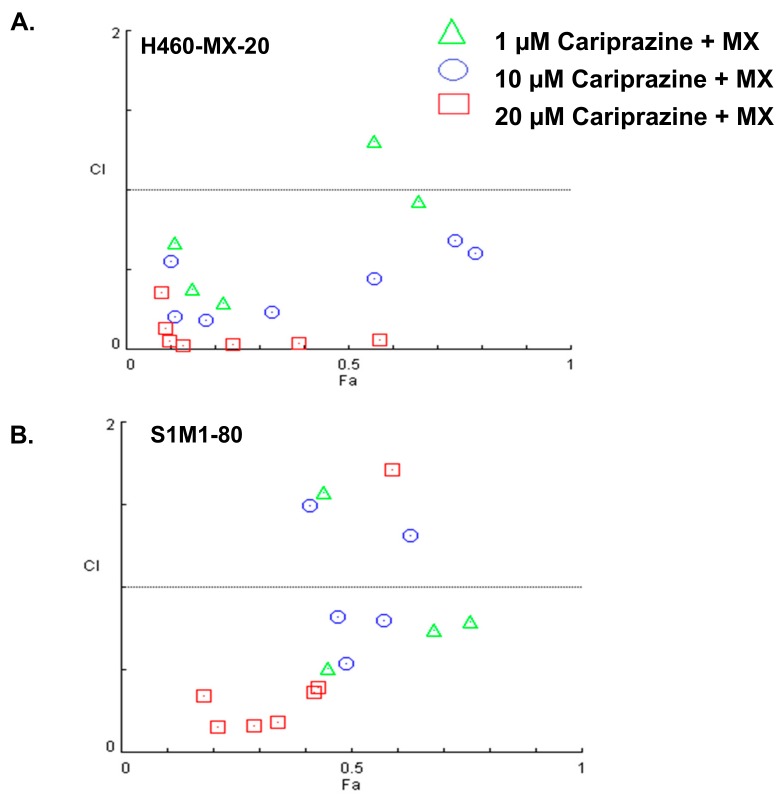
The combination index values for the combination of 1, 10 or 20 μM of cariprazine, respectively, with 0.1, 0.3, 1, 3, 10, 30, or 100 μM of MX in (**A**): H460-MX20 cancer cells and (**B**) S1M1-80 cancer cells. The Fa values range from 0 to 1. CI < 1, synergism; CI = 1, additive effect and CI > 1, antagonism. The data represent the mean ± SD of three independent experiments in triplicate.

We used the method of Chou and Talaly to determine if cariprazine produced synergistic efficacy in combination with MX in H460-MX20 cells and S1M1-80 cells. Based on the combination index (CI) ranges values, the CI values were determined from a Fa range of 0 to 1. In H460-MX20 cells, the combination of 1 μM of cariprazine with 10 and 30 μM of MX produced a synergistic effect (CI = 0.3–0.7; Figure 4A), whereas the combination of 1 μM of cariprazine with 1 or 100 μM MX produced a moderate synergistic effect (CI = 0.7–0.85; Figure 4A). The combination of 10 μM of cariprazine with 3 μM of MX, as well as 10 and 30 μM of MX combined with 10 μM of cariprazine, produced strong synergism (CI = 0.1–0.3; Figure 4A). The combination of 10 μM of cariprazine and 0.1–1 μM of MX, as well as 100 μM of MX combined with 10 μM of cariprazine, produced a synergistic effect, but to a lesser extent than the previous combinations (CI = 0.3–0.7; Figure 4A). The combination of 20 μM of cariprazine and 0.1–1 μM of MX produced a very strong synergistic effect (CI < 0.1; Figure 4A). The combination of 20 μM of cariprazine and 30–100 of MX produced a strong synergistic effect (CI = 0.1–0.3; Figure 4A).

In S1-M180 cells, the combination of 1 μM of cariprazine and 1–30 μM of MX produced a synergistic effect (CI = 0.3–0.7; Figure 4B). However, the combination of 1 μM of cariprazine with 1 or 100 μM of MX produced moderate antagonism (CI = 1.2–1.45; Figure 4B). The combination of 10 μM of cariprazine and 3–30 μM of MX produced a synergistic effect (CI = 0.3–0.7; Figure 4B). However, the combination of 10 μM of cariprazine with 1 or 100 μM of MX produced moderate antagonism (CI = 1.2–1.45; Figure 4B). The combination of 20 μM of cariprazine and 3–30 μM of MX produced a strong synergistic effect (CI = 0.1–0.3; Figure 4B). Moreover, the combination of 20 μM of cariprazine and 0.3, 1 or 100 μM of MX produced a synergistic effect (CI = 0.3–0.7; Figure 4B). In contrast, the combination of 20 μM of cariprazine and 0.1 μM of MX produced moderate antagonism (CI = 1.2–1.45; Figure 4B).

### 2.3. Cariprazine Decreases the Protein Expression Levels of the ABCG2 Transporter Protein

**Figure 5:**
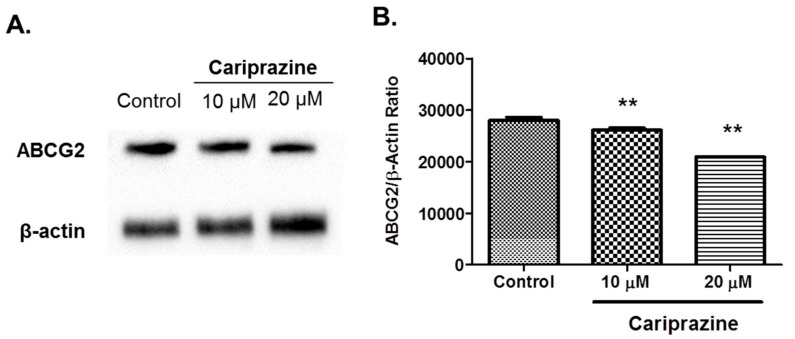
The effects of cariprazine on the expression level of the ABCG2 transporter protein. (**A**) H460-MX20 cells were incubated with 10 or 20 μM cariprazine, respectively. (**B**) The columns represent the mean of the western blot quantification values. The error bars represent the SEM. At least two additional experiments showed similar results to the representative figure results. ** *p* < 0.01.

Western blot assays were performed to determine the mechanism by which cariprazine modulates MDR and increases in the efficacy of MX in ABCG2 overexpressing H460-MX20 cells. The incubation of H460-MX20 cells with 10 or 20 μM of cariprazine produced a significant downregulation of the ABCG2 transporter compared to the controls, with the effect at 20 μM being significantly greater than that of 10 μM (Figure 5).

### 2.4. Cariprazine Inhibits the Efflux Function of the ABCG2 Transporter

**Figure 6:**
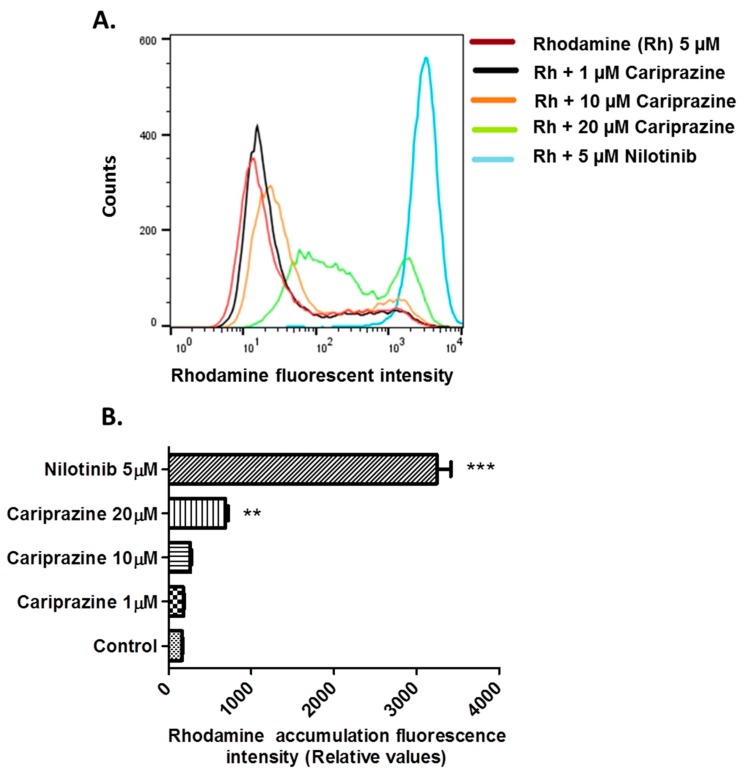
The effect of cariprazine, and nilotinib on the intracellular accumulation of rhodamine 123 in ABCG2 overexpressing S1M1-80 cells. (**A**) The accumulation of rhodamine 123 was increased in a dose-dependent manner in S1M1-80 cells incubated with cariprazine. Red filled, control; black, 1 μM; orange, 10 μM; green, 20 μM; blue, nilotinib, 5 μM. (**B**) Rhodamine 123 levels were expressed as the units of mean fluorescent intensity. The data shown as means ± SD of triplicate determinations. ** *p* < 0.05; *** *p* < 0.001 vs. the control.

To further elucidate the mechanisms that mediate cariprazine’s reversal of ABCG2-mediated MDR, we measured the effect of cariprazine on the intracellular accumulation of the fluorescent dye Rhoda mine 123, a substrate for ABC transporters, in ABCG2 overexpressing S1M1-80 cells. The incubation of S1M-80 cells with 1 or 10 μM of cariprazine did not significantly alter the intracellular accumulation of Rhodamine 123 compared to cells incubated with vehicle (Figure 6). In contrast, Rhodamine 123 accumulation was significantly increased by 20 μM of cariprazine compared to the control and the 1 and 10 μM concentrations of cariprazine (Figure 6). In addition, as previously reported [21], nilotinib (5 μM), an inhibitor of the ABCB1 and ABCG2 efflux function, significantly increased the intracellular accumulation of rhoda mine compared to controls and 1, 10, or 20 μM of cariprazine (Figure 6). However, the increase in the intracellular accumulation of Rhodamine level in S1M-80 cells incubated with 1–20 μM of cariprazine was of a lower magnitude compared to the intracellular accumulation of Rhodamine in S1M-80 cells incubated with 5 μM of nilotinib.

### 2.5. Molecular Docking Analysis of the Interaction of Cariprazine with A Human ABCG2.

**Figure 7:**
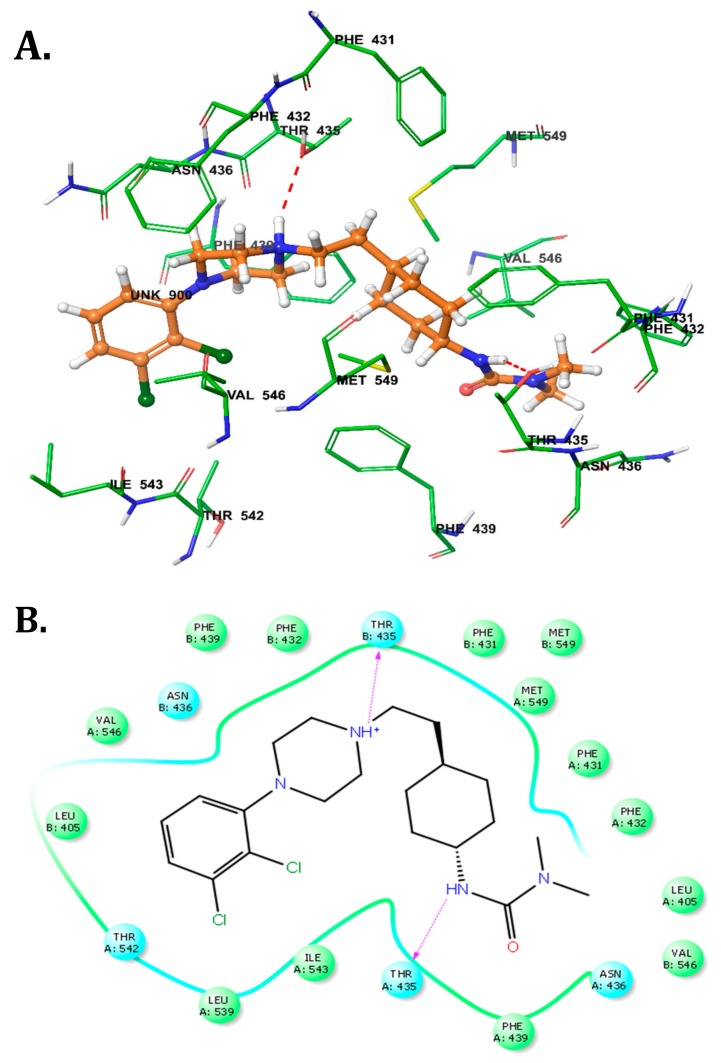
Model for the binding of cariprazine with ABCG2 transporter. (**A**) XP-Glide predicted binding mode of cariprazine in drug binding cavity of human ABCG2 transporter (PDB ID: 6ETI). Important amino acids are depicted as sticks with the atoms colored as carbon = green, hydrogen = white, nitrogen = blue, oxygen = red. The ligand cariprazine is shown with the same color scheme as above except for the carbon atoms, represented in orange and the chlorine atoms represented in dark green. The red dotted lines represent hydrogen bond. (**B**) A schematic diagram of the protein–ligand interaction is shown for cariprazine. Blue circles depict polar amino acids and green circles depict hydrophobic amino acids. The purple arrows depict side chain donor/acceptor interactions.

Molecular docking studies were performed to determine the binding mode of cariprazine to the drug binding cavity of ABCG2 (PDB ID: 6ETI), as described in the experimental section [22]. Figure 7 illustrates the XP Glide predicted docked conformation of cariprazine at the drug binding cavity 1 located in vicinity to two-fold symmetry axis of ABCG2. The 2,3-dichloro phenyl moiety linked to the piperazinyl ring in cariprazine is favorably placed in a hydrophobic pocket flanked by the amino acid residues Leu405, Leu539, Thr542, Ile543, and Val546, thereby enabling hydrophobic interactions. The piperizinyl ring of cariprazine is positioned hydrophobic channel lined by residues Phe432, Phe439 and Val546 and is further stabilized by hydrogen bonding interaction between quaternary NH^+^ of piperizinyl ring ring and Hydroxyl ‘Oxygen’ of residue Thr435 of chain B (N^+^-H•••O-Thr435 (B), 2.54 Å). This interaction is significant, as a similar interaction with this residue is observed for the ABCG2 modulator fumitremorgin C-related inhibitor Ko143 (PDB ID: 6FEQ) [22]. The 3-cyclohexyl-1,1-dimethylurea moiety attached to the piperazinyl moiety by an ethylene linker and isalso stabilized through hydrophobic contacts with amino acid side chains Leu405, Phe431, Phe432, Phe439, Val546, and two Met549 (Chain A & B). The ‘NH’ group of the 1,1-dimethylurea also forms a hydrogen bond with the Hydroxyl oxygen atom of Thr435 (N-H•••O-Thr435 (A), 2.12 Å). The Glide XP score of cariprazine was −6.515.

## 3. Discussion

This study is the first to report that cariprazine, a D_3_/D_2_ receptor partial agonist, increases the efficacy of MX (an ABCG2 substrate) in the ABCG2 overexpressing cancer cell lines H460-MX20 and S1M1-80. Specifically, cariprazine, at 1, 10, and 20 μM, significantly increase the efficacy of MX in H460-MX20, and S1M1-80 by significantly reducing the IC_50_ value of MX in a concentration-dependent manner. Conversely, the sensitivity of the parental cell lines, H460 and S1, which do not overexpress ABCG2 transporters, to MX, were not significantly enhanced by cariprazine. The concentrations of cariprazine used (1–20 μM) were all below concentrations that decreased cell viability, indicating that the effects of cariprazine on the cancer cell lines was not due to it producing cytotoxicity. Thus, cariprazine significantly and selectively modulates MDR in ABCG2 overexpressing cancer cells. However, additional studies should be conducted to determine whether cariprazine is a reversal compound that specifically targets the ABCG2 protein.

Cariprazine, at concentrations of 1, 10 or 20 μM, significantly reduced the fold-resistance (e.g., the magnitude of resistance in the ABCG2-overexpressing cells to MX compared to the parental cell lines) in H460-Mx20 cells from 28 to 20, 28 to 5, and 28 to 1, respectively. Similarly, cariprazine, at concentrations of 1, 10 or 20 μM, significantly reduced the fold-resistance in S1M1-80 from 93 to 53, 93 to 20, and 93 to 1.3, respectively. H460-MX20 cells treated with a combination of cariprazine and 0.3 μM of MX showed a significant reduction in cell number and significant changes in the morphological features such as progressive shrinkage of the cells, loss of membrane integrity, and cell lysis, compared to H460-MX20 cells incubated with 0.3 μM of MX alone. Similarly, S1M1-80 cells incubated with a combination of cariprazine and 10 μM of MX showed a significant reduction in cell number and significant changes in the morphological features such as progressive shrinkage of the cells, loss of membrane integrity, and cell lysis as compared with S1M1-80 cells incubated with 10 μM of MX alone. The above findings are congruent with the molecular docking data indicating that cariprazine interacts with the ABCG2 transporter. Nilotinib, at 5 μM, significantly reduces the fold-resistance of H460-MX20 cells to MX [23]. Our results are consistent with previous studies indicating that nilotinib significantly reduces the fold-resistance of HEK293-R2 cells to MX and doxorubicin (DOX) as compared to FTC (an ABCG2 modulator) [21]. In addition, nilotinib produced cell shrinkage, fragmentation into membrane-bound apoptotic bodies and rapid phagocytosis by neighboring cells. Our findings showed that cariprazine has a lower potency than nilotinib in reversing ABCG2-mediated MDR.

Previous studies over the last four decades have characterized ABC transporter modulators [24]. However, none of these compounds received food and drug administration (FDA) approval due to a lack of efficacy, toxicity, problematic drug-drug interactions, and problematic pharmacokinetic properties (PK) [14]. For example, in vitro studies showed that FTC was a potent ABCG2 modulator, enhancing the efficacy of many conventional chemotherapeutic drugs, such as MX, DOX, and topotecan [25]. However, in vivo studies indicated that FTC was extremely toxic, thus precluding its use in humans [14]. Previous studies have shown that D_3_ receptor antagonists, which have structural homology to tyrosine kinase inhibitors (TKIs) like nilotinib, which are known ABCG2 modulators, have efficacy in cancer cells overexpressing ABCG2 transporters [19]. Predominantly through hydrophobic interactions, it has been suggested that the D_3_ receptor antagonists NGB2904, PG01037, SB277011A and U99194A bind at the substrate-binding site of the ABCG2 transporter [19]. Furthermore, the aforementioned D_3_ receptor antagonists significantly reversed ABCG2-mediated MDR in both lung and colon cancer cell lines overexpressing ABCG2 transporters, by inhibiting the efflux function of ABCG2 transporters and down regulating the expression levels of ABCG2 transporters [19]. Based on the structural homology to the aforementioned modulators, we hypothesized that cariprazine could inhibit the ABCG2 transporter and/or be a substrate. The molecular docking simulations predicted that cariprazine was stabilized into the transmembrane domain of ABCG2 via hydrophobic interactions. These results suggest that cariprazine may inhibit competitively inhibit the ABCG2 transporter, although this remains to be determined. However, additional mechanistic studies, such as the ATPase assay, photo-affinity labeling, and membrane vesicle uptake assays should be done as the in-silico studies alone cannot ascertain if cariprazine binds to the ABCG2 substrate sites. Previously, we reported that the rank order of the docking scores for the D_3_ receptor antagonists NGB2904, PG01037 SB277011A and U99194A was similar to the rank order of potency for reversal of ABCG2-mediated resistance [19]. The XP score obtained from our docking analysis for cariprazine was −6.515, which was lower than the docking scores of D_3_ dopamine receptor antagonists NGB2904, PG01037, SB277011A and U99194A, which were −9.704, −9.299, −9.068 and −6.525, respectively. These results may explain why cariprazine was less efficacious in the reversal of resistance to MX in the MX-resistant cell lines as compared with D_3_ dopamine receptor antagonists. However, it is important to note that in vitro, cariprazine’s K_i_ = 0.085 nM for brain dopamine D_3_ receptors [26]. Thus, cariprazine has structural features that allow it to bind with high affinity to DA D_3_ receptors, but not to the ABCG2 protein.

There is a substantial body of literature indicating that specific drug combinations for cancer treatment have distinct advantages compared to use of single chemotherapeutic drugs [27,28]. Our in vitro results indicated that cariprazine produces a concentration-dependent, synergistic effect when combined with MX. In H460-MX20 cells, the combination of 20μM of cariprazine and 0.1–100 μM of MX produced efficacy that ranged from strong to very strong synergism. Similarly, the combination of 20 μM of cariprazine, in S1M1-80 cells, with 0.1–100 μM MX produced efficacy that ranged from synergism to strong synergism, although 20 μM of cariprazine and 0.1 μM of MX produced a moderate antagonism of the efficacy of MX. In both H460-MX20 and S1M1-80 cells, the combination of 10 μM of cariprazine and 0.1–100 μM of MX produced synergism to strong synergism. However, the combination of 10 μM cariprazine, in S1M1-80 cells, with 1 or 100 μM of MX produced a moderate antagonism of the efficacy of MX. Moreover, the combination of 1 μM cariprazine and 0.1–100 μM of MX, in both H460-MX20 and S1M1-80 cells, produced synergism to moderate synergism of the efficacy of MX. However, 1 μM of cariprazine produced a moderate antagonism of the efficacy of 1 or 100 μM of MX in S1M1-80 cells. These variations may result from lower concentrations of the cariprazine, which significantly augmented MX efficacy, thereby yielding less variable results (i.e., synergism only). Intriguingly, the combination of cariprazine and MX produces efficacy ranging from synergism to strong synergism as compared with the efficacy of the combination of the D_3_ receptor antagonists NGB2904, PG01037, SB277011A, U99194A and MX, which ranged between strong to very strong synergism [19]. The above findings corroborate the MTT and docking studies results, explaining the differences in the efficacy of cariprazine as compared to the D_3_ receptor antagonists NGB2904, PG01037 and PG01037.

Our results indicated that cariprazine surmounted ABCG2-mediated resistance, in part, by downregulating the levels of the ABCG2 protein. Previously, it has been reported that a decrease in the efficacy of chemotherapeutic drugs in primary cell cultures from cancer patients are due, to some extent, to the high expression of intrinsic MDR transporters, e.g., ABCG2, and their overexpression induced by long-term exposure to chemotherapeutic drugs [29]. It is well established that the downregulation of the protein expression of specific ABC transporter is one of the major mechanism responsible for reversing ABC-mediated MDR [30]. For example, the downregulation of the ABCG2 transporter was the major mechanism by which toremifene [31], YHO-13177 [32], artesunate [33], and HM015K [34] and the DA D_3_ antagonists NGB2904, PG01037, SB277011A and U99194A [19], significantly increased the in vitro efficacy certain anticancer drugs that are substrates for the ABCG2 transporter. Western blot experiments were conducted to investigate the mechanism by which cariprazine circumvents MDR. Our results indicated that the incubation of the ABCG2 overexpressing cancer cells, H460-MX20, with 10 or 20 μM cariprazine for 24 h significantly decreased ABCG2 protein levels in in a concentration dependent-manner. Previous studies have shown that the expression of the ABCG2 protein in various cancer cell lines is mediated by the activation of c-jun N-terminal kinase, mitogen-activated protein kinases, phosphate and tensin homolog, epidermal growth factor receptor and human epidermal growth factor (EGFR) [35]. The in vitro expression of ABCG2, at the transcription and at post-translational level, was significantly decreased in isogenic U87MG human glioblastoma cell lines by PD153035 (an EGFR antagonist) [36]. The cariprazine-induced decrease in ABCG2 protein levels could result from the rapid internalization and degradation via lysosomes and/or proteasomes. Current evidence suggests that a number of trans-acting elements, including hypoxia inducible factor 1α, estrogen receptor and peroxisome proliferator-activated receptor regulate *BCRP* gene transcription could play a role in the expression of the ABCG2 gene [37]. Cariprazine could decrease protein expression levels by decreasing (1) ABCG2 gene transcription and/or (2) mRNA stability. The mechanism(s) by which cariprazine downregulates ABCG2 protein expression remains to be elucidated.

One of the major findings of this study is that cariprazine, in a concentration-dependent manner, significantly increased Rhodamine 123 accumulation, an ABCG2 substrate, by preventing efflux in S1M1-80 cells. Additionally, as previously reported [25], 5 μM of nilotinib also significantly increased the intracellular levels of Rhodamine 123 by inhibiting the efflux function of the ABCG2 transporter. However, the magnitude of the cariprazine-increase in Rhoda mine 123 levels in the S1-M1-80 cells was about four times lower than that for 5 μM of nilotinib. These results, in combination with the docking analysis data for cariprazine, suggest that these compounds surmount ABCG2-mediated resistance to MX by inhibiting the efflux function of the ABCG2 transporter. Our results with cariprazine are consistent with those reported for other compounds, such as Ko143 [38], novobiocin [39], imatinib [40], and NGB2904, PG01037, SB277011A and U99194A [19], which have been shown to modulate ABCG2-mediated MDR by inhibiting ABCG2 efflux function in cell lines overexpressing ABCG2 transporters. However, further studies must be done to ascertain whether cariprazine modulates the efflux function of ABCG2 transporter through direct binding to the efflux site of ABCG2 transporter mechanism or through other mechanisms.

## 4. Materials and Methods

### 4.1. Reagents

Cariprazine hydrochloride was purchased from Advanced Chemblock Inc. (Burlingame, CA, USA). Nilotinib was purchased from Sigma-Aldrich (St. Louis, MO, USA). Mitoxantrone was purchased from Selleck Chemicals (Farmingdale, NY, USA). Rhodamine 123 fluorescent dye was purchased from Marker Gene Technologies Inc. (Eugene, OR, USA). 3-(4,5-dimethylthiazol-2-yl)-2, 5-diphenyltetrazolium bromide (MTT) was purchased from Calbiochem EMD Millipore (Billerica, MA, USA). Dulbecco’s modification of Eagle’s medium (DMEM) and 0.25% trypsin + 2.2 Mm ethylenediaminetetraacetic acid (EDTA), phosphate buffered saline (PBS without calcium or magnesium), and DMEM (phenol red-free) were purchased from Mediatech, Inc. (Corning subsidiaries, Manassas, VA, USA). Fetal Bovine Serum (FBS) was purchased from Bio Fluid Technology Inc. (Bryn Mawr, PA, USA). Monoclonal antibody against beta-actin (β-actin) and anti-mouse secondary antibody were purchased from Cell Signaling Technology (Danvers, MA, USA). Bicinchoninic acid (BCA) solution and copper solution were purchased from G-Biosciences (St. Louis, MO, USA). Mouse monoclonal antibody (BXP-21) for the ABCG2 transporter protein was purchased from Novus Biologicals (Littleton, CO, USA). Mini-Protean^®^TGX™ precast Gels, Clarity™ and Clarity Max™ Western ECL Blotting Substrates were purchased from Bio-Rad Laboratories (Hercules, CA, USA). Polyvinylidene difluoride (PVDF) membrane was purchased from Thermo Fisher Scientific (Waltham, MA, USA). Nonfat dry milk was purchased from Cell Signaling (Danvers, MA, USA). Tween-20 was purchased from Fisher Scientific (Springfield Township, NJ, USA).

### 4.2. Cell Lines and Cell Culture

The mitoxantrone-resistant colon cancer cells, S1M1-80 (overexpressing ABCG2 with the G482 mutation), were grown as adherent monolayers in culturing flasks with DMEM media supplemented with 10% FBS and 1% streptomycin/penicillin in a humidified incubator with 5% CO_2_ at 37 °C. The cells were maintained with 80 µM of mitoxantrone in the medium. NCI-H460 human lung non-small cell carcinoma cells were grown as adherent monolayers in culturing flasks and selected for overexpression of ABCG2 by maintenance in DMEM/10% FBS supplemented with 1 nM mitoxantrone. The above cells were a gift from the late Dr. Gary Kruh (University of Chicago, Chicago, IL, USA).

### 4.3. Cell Cytotoxicity through Colororimeteric MTT Assay

Cell viability was determined using the (3-(4,5-dimethylthiazol-2-yl)-2,5-diphenyltetrazolium bromide) (MTT) as previously described [41]. Both resistant and parental cancer cell lines were resuspended in DMEM after harvesting the cells with 0.25% trypsin + 2.2 mM EDTA. The cells were seeded evenly (160 μL/well) at a density of 5000 cells/well in 96 well plates. Cells were incubated with serial dilutions of the anticancer drug, mitoxantrone (MX, 0, 0.1, 0.3, 1, 3, 10, 30, and 100 μM) during the second day of the experiment, followed by various concentrations of cariprazine hydrochloride and 5 μM of nilotinib (20 μL/well). The MTT dye (4 mg/mL) was added after 72 h of incubation and incubated with the cells for an additional 4 h at 37°C. Following incubation, the media was discarded, and the formed formazan crystals were dissolved by adding 100 µL of DMSO to each well. The absorbance was determined using a SpectraMAX iD3^®^ Multi-mode microplate reader (Sunnyvale, CA, USA) at 570 nm. The IC_50_ was determined using the Bliss method [42], which is based on the change in the percentage of viable cells after the addition of chemotherapeutic drugs, with or without the reversal compounds. The magnitude of the drug resistance (the resistance-fold) was determined by dividing the IC_50_ obtained in the resistant cancer cells (with or without the reversal compounds) by the IC_50_ of the nonresistant, parental cancer cells.

### 4.4. Cell Morphological Analysis

The cells were photographed for each triplicate compound and vehicle using an EVOS Fluorescence Cell Imaging System^®^. Morphological changes were observed 72 h after incubation with the chemotherapeutic drug MX alone, or the combination of the chemotherapeutic drug with cariprazine. The images were compared in a double-blind fashion for the different concentrations of the tested compound at different time points for cell size, shape and numbers. A representative figure is shown for each cell line.

### 4.5. Protein Estimation: Cell Lysate Preparation and Bicinchoninic Acid (BCA) Analysis

H460-MX20 cells, in T25 flasks, were incubated with 10 or 20 μM of cariprazine for 24 h. The media was aspirated, and the cells were rinsed with 5 mL of PBS drop by drop, followed by a gentle shaking and aspiration of PBS. Two milliliters of PBS were added three times to the cells, which were collected for lysis by using a cell scraper to detach the cells. During the transfer of the cells from flasks to 15 mL tubes, they were kept on ice. The tubes were centrifuged at 1500 rpm for 3 min and the supernatant was discarded and the white pellets were kept for the cell lysis step. The cytosolic fraction was extracted by the addition of 400 μL of ice-cold lysis buffer A [1M Hepes-KOH (pH 7.9), 45 mM MgCl2, 300 mM KCL, 50 mM dithiothreitol (DTT), 100 mM phenylmethylsulfonyl fluoride (PMSF), and 3 mL of a protease inhibitor cocktail (ThermoScientific, Waltham, MA, USA). to each sample. The sample pellets were resuspended by vortexing and transferred to eppendorf tubes. The cell homogenates were kept on ice for 15 min and 23 μL of NP40, which is a nonionic surfactant that solubilizes the plasma membrane and the internal membranous organelles, was added to each sample for 2 min to facilitate the isolation and the purification of functional membrane protein complexes in two-dimensional gel electrophoresis. The samples were centrifuged at 13,000 rpm for 1 min and the supernatant layer, containing membranous proteins, was aspirated and kept at −20 °C until analysis. The concentrations of the proteins in the samples were determined using the BCA assay. A standard curve, using 8 different concentrations of bovine serum albumin, was prepared. The working solution used was composed of BCA solution and copper solution in a ratio of 50 parts BCA: 1-part copper solution. Two hundred microliters of the working solution were added to the samples and standard wells. After incubating the plate at 37 °C, the absorbance was measured at 562 nM using SpectraMAX iD3® Multi-mode microplate reader (Sunnyvale, CA, USA).

### 4.6. Western Blot Analysis

Protein samples were analyzed by loading equivalent amounts (20 μg) onto 7.5% SDS-polyacrylamide gels using the Mini-PROTEAN® Tetra Cell assay from Bio-Rad (Hercules, CA, USA). The proteins were separated based on their molecular weight and then transferred to a PVDF membrane, which was previously activated using methanol. In order to prevent nonspecific binding, the membrane was incubated 1 h with 5% nonfat milk in TBS-T (10× Tris buffered saline (TBS) [24.2 g Tris base, 80 g NaCl, 1N HCl, 38 mL] containing 0.1% Tween-20) before adding the primary BXP-21 anti-ABCG2 antibody (1:1000). The membrane was incubated with the primary antibody overnight at 4 °C with gentle shaking. After overnight incubation, the membrane was washed three times with TBS-T washing buffer. Before adding the horseradish peroxide (HRP)-linked anti-mouse secondary antibody (1:3000; cell signaling), the membrane was incubated with blocking buffer. Subsequently, the membrane was incubated with the secondary antibody for 90 min at room temperature and washed 3 times with TBS-T buffer. The Western ECL blotting substrates (Western peroxide reagent, and clarity Western luminol enhancer) were added in a 1:1 ratio before the blots were read using a Molecular imager® ChemiDoc™ XRS+ from BIO RAD (Hercules, CA, USA). β-actin was used as a housekeeping control for protein samples. Image quantification analysis of Western blots was done using either Image Lab or Image J software (version 1.41, NIH, Bethesda, Rockville, MD, USA).

### 4.7. Rhodamine 123 Accumulation Assay

The ABCG2 overexpressing S1M1-80 cells were seeded at a density of 5 × 10^5^ cells per ml in six-well plates and allowed to attach overnight in phenol red-free media. The cells were incubated with 1, 10 or 20 μM of cariprazine or 5 μM of nilotinib for 1 h. Subsequently, 5 μM of Rhoda mine 123 was added and the cells were further incubated for 1 h. The cells were rinsed twice with ice-cold, complete phenol red-free media. The cells were harvested and centrifuged at 400× *g* for 5 min, washed twice with ice-cold PBS, and dispersed in 1 mL of PBS. Cellular Rhoda mine 123 accumulation was analyzed in 10,000 events per 1 mL of the sample using a flow cytometer from BD Biosciences FACS Calibur (San Jose, CA, USA) and the data was captured using C Flow^®^ Plus software. The data were further analyzed using either FCS Express 5 Plus (Glendale, CA, USA) and/or Flow Jo V10 software (Ashland, OR, USA).

### 4.8. The Effect of Cariprazine on the Efficacy of MX

In order to determine if cariprazine can increase the efficacy of anticancer drugs, such as MX, the combination index (CI) was determined based on the Chou and Talalay method [43]. The CI represents a quantitative measure of the magnitude of the additive, synergistic or antagonistic drug effects [43]. The data were analyzed using CompuSyn software (CompuSyn, NJ). The CI values are interpreted as follows: antagonism, CI > 1; addition, CI = 1; and synergism, CI < 1. The fraction-affected value Fa corresponds to the percent of cells that survive, whereas Fa = 0 when the percentage of cells surviving = 100% and Fa = 1 when the percentage of cells surviving is equal to 0%.

### 4.9. Molecular Docking Studies

#### 4.9.1. Ligand Structure Preparation

The structure of cariprazine was constructed using the builder module of Maestro v9.3.5 and subsequently energy minimized by Macromodel program v9.9 (Schrödinger, Inc., New York, NY, USA, 2012), using the OPLSAA force field with the steepest descent, followed by using the truncated Newton conjugate gradient protocol. The low-energy 3D structures of cariprazine were generated by LigPrep v2.5 and the parameters were defined based on different protonation states at physiological pH ± 2, and all possible conformations were filtered with a maximum relative energy difference of 5 kcal/mol to exclude redundant conformers. The ligand structures obtained from the LigPrep v2.5 run was further used for generating 100 ligand conformations for each protonated structure using the default parameters of mixed torsional/low-mode sampling. The output conformational search (Csearch) file, containing 100 unique conformers of cariprazine, was used as input for docking simulations at the binding site of the human ABCG2 transporter protein.

#### 4.9.2. Protein Structure Preparation

The X-ray crystal structure of ABCG2 in complex with fumitremorgin C-related inhibitor MZ29 (PDB ID:6ETI) reported by Jackson et al. [22], was utilized for the molecular docking studies. The protein was optimized using the ‘Protein Preparation Wizard’ workflow implemented in the Schrödinger molecular modeling suite (Schrödinger, Inc., New York, NY, USA, 2018-1). This optimization includes adding hydrogen atoms, assigning correct bond orders and building disulfide bonds. The protonation states of all of the ionizable residues were predicted by PROPKA provided in the protein preparation wizard. An optimized structure model was energy minimized (only hydrogen atoms) using the OPLS2005 force field. The refined protein model was used to generate receptor grid based on the centroid of co-complexed ligand MZ29 using default settings.

#### 4.9.3. Docking Protocol

The diverse conformational library of cariprazine was docked at the generated grid using the “Extra Precision” (XP) [44] model of Glide program v7.8 [45] (Schrödinger, Inc., New York, NY, USA, 2018-1) with the default functions. The best-docked conformations of cariprazine as established by high XP GScore were chosen and used for further analysis. All computations were carried out using a Core i5 processor workstation with Windows 10 operating system.

## 5. Conclusions

Our results suggest that cariprazine significantly reverses ABCG2-mediated MDR and re-sensitizes both lung and colon cancer cell lines overexpressing ABCG2 transporters to substrate antineoplastic drugs like MX by inhibiting the efflux function of the transporter and downregulating the expression levels of ABCG2 transporter. The significant in vitro reversal effect (i.e., increase in MX efficacy) produced by cariprazine was concentration-dependent and occurred at in vitro concentrations significantly below those that decreased cell viability. Additionally, cariprazine significantly inhibited the intracellular ABCG2 efflux function. Furthermore, cariprazine also produced a significant and concentration-dependent decrease in ABCG2 protein levels. The combination of specific concentrations of cariprazine and the ABCG2 transporter substrate, MX, produced a synergistic increase in the efficacy of MX. Our results, provided they can be extended to humans, suggest that cariprazine could be used in combination with FDA approved anticancer drugs, which are ABCG2 substrates, to increase the likelihood of overcoming cancers that overexpress ABCG2 transporters. Finally, further mechanistic studies, such as photo affinity labeling, ATPase assay, and membrane vesicle uptake assays must be done to ascertain the interaction of cariprazine with the ABCG2 transporter.

## Figures and Tables

**Table 1:** The effect of cariprazine and nilotinib on the cytotoxicity of MX in ABCG2-overexpressing H460-MX20 cells.

Treatment	IC_50_ ± SEM (µM) ^a^			
	H460	FR ^b^	H460-MX20	FR ^b^
**Mitoxantrone**	0.22 ± 0.01	1	6 ± 0.1	28
**+Cariprazine 1 µM**	0.2 ± 0.09	1	4.36 ± 0.3 *	20
**+Cariprazine 10 µM**	0.2 ± 0.06	1	1 ± 0.5 **	5
**+Cariprazine 20 µM**	0.2 ± 0.02	1	0.2 ± 0.04 **	1
**+Nilotinib 5 µM**	0.2 ± 0.04	1	0.4 ± 0.01 **	2

^a^ The IC_50_ data is presented in the two columns, H460 and H460-MX20, for the two cell lines as the mean ± SEM (standard error of the mean) of at least three experiments conducted in triplicate. ^b^ Fold-resistance (FR) is presented in the adjacent columns, calculated as described in the materials and methods. The percentage of surviving cells was determined using the MTT assay. Statistical significance was determined in comparison to the control condition (MX alone) * *p* < 0.05; ** *p* < 0.01.

**Table 2:** The effect of cariprazine and nilotinib on the cytotoxicity of MX in ABCG2-overexpressing S1M1-80 cells.

Treatment	IC_50_ ± SEM (µM) ^a^			
	S1	FR ^b^	S1M1-80	FR ^b^
**Mitoxantrone**	0.5 ± 0.01	1	46 ± 0.57	93
**+Cariprazine 1 µM**	0.5 ± 0.02	0.9	26.4 ± 0.12 ***	53
**+Cariprazine 10 µM**	0.43 ± 0.01	0.9	10 ± 0.09 ***	20
**+Cariprazine 20 µM**	0.6 ± 0.01	1	0.66 ± 0.007 ***	1.332
**+Nilotinib 5 µM**	0.44 ± 0.02	0.9	0.44 ± 0.01 ***	0.888

^a^ The IC_50_ data is presented in the two columns, S1 and S1-M180, for the two cell lines as the mean ± SEM (standard error of the mean) of at least three experiments conducted in triplicate. ^b^ Fold-resistance (FR) is presented in the adjacent columns, calculated as described in the materials and methods. The percentage of surviving cells was determined using the MTT assay. Statistical significance was determined in comparison to the control condition (MX alone) *** *p* < 0.001.
